# Pandemic (H1N1) 2009 Virus in 3 Wildlife Species, San Diego, California, USA

**DOI:** 10.3201/eid1704.101355

**Published:** 2011-04

**Authors:** Mark D. Schrenzel, Tammy A. Tucker, Ilse H. Stalis, Rebecca A. Kagan, Russell P. Burns, Amy M. Denison, Clifton P. Drew, Christopher D. Paddock, Bruce A. Rideout

**Affiliations:** Author affiliations: San Diego Zoo Global, Escondido, California, USA (M.D. Schrenzel, T.A. Tucker, I.H. Stalis, R.A. Kagan, R.P. Burns, B.A. Rideout);; Centers for Disease Control and Prevention, Atlanta, Georgia, USA (A.M. Denison, C.P. Drew, C.P. Paddock)

**Keywords:** Viruses, influenza, pandemic (H1N1) 2009, respiratory infections, California, zoological gardens, wildlife, reservoirs, H1N1, letter

**To the Editor:** The influenza A pandemic (H1N1) 2009 virus rapidly created a global pandemic among humans and also appears to have strong infectivity for a broad range of animal species (*1**–**3*). The virus has been found repeatedly in swine and has been detected in a dog, cats, turkeys, and domestic ferrets and in nondomestic animals, including skunks, cheetahs, and giant anteaters (*2**–**4*). In some cases, animal-to-animal transmission may have occurred, raising concern about the development of new wildlife reservoirs (*2*).

In 2009, the first recognized occurrence of pandemic (H1N1) 2009 in southern California in April was followed by a surge of cases during October through November (*4*). During this time, respiratory illness developed in a 12-year-old male American badger (*Taxidea taxus taxus*), a 19-year-old female Bornean binturong (*Arctictis binturong penicillatus*), and a 7-year-old black-footed ferret (*Mustela nigripes*) housed in a San Diego zoological garden.

The 3 affected animals had clinical signs that included lethargy, inappetance, dyspnea, nasal discharge, and coughing. The severity of disease in the badger and binturong necessitated euthanasia; the ferret recovered with antibiotic and fluid therapy. Postmortem examination revealed bronchopneumonia with diffuse alveolar damage in the badger and interstitial pneumonia with diffuse alveolar damage in the binturong. Bacterial cultures and Gram stains of affected lung samples were negative.

Molecular analyses for several groups of viruses, including *Herpesviridae*, *Paramyxoviridae*, *Adenoviridae*, and all influenza A viruses, were performed on frozen lung samples from the badger and binturong and on frozen conjunctival and pharyngeal swabs from the ferret. Results of PCRs specific for segments of influenza A nucleoprotein, matrix protein, hemagglutinin, and neuraminidase genes were positive in samples from all 3 animals, and DNA sequencing of amplicons identified the viruses as pandemic (H1N1) 2009. Influenza A virus was not detected in samples from the ferret after it recovered. Results of PCRs for all other viruses were negative. Immunohistochemical evaluation of lung samples from the badger for antigens of influenza A virus (*5*) showed rare staining in bronchiolar epithelial cells (Figure).

**Figure:**
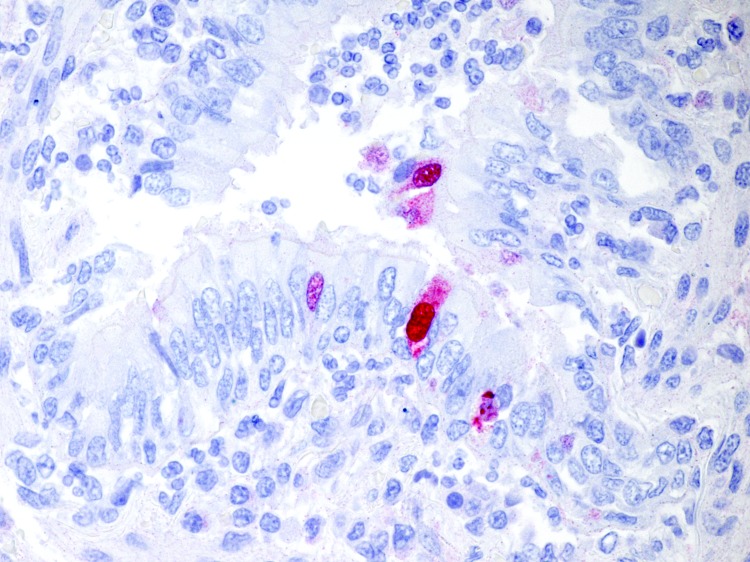
Lung section from an American badger showing immunohistochemical staining (red chromogen) specific for the pandemic (H1N1) 2009 virus within the nucleus and cytoplasm of bronchiolar epithelial cells and concurrent inflammatory cell infiltrates; hematoxylin counterstain. Original magnification ×158.

Respiratory disease in all 3 affected animals seemed to be caused by pandemic (H1N1) 2009 virus. The badger and binturong were generally healthy, no other pathogens were detected, and pulmonary lesions were consistent with influenza pneumonia. In these animals, pandemic (H1N1) 2009 infection was especially aggressive, resulting in irreversible disease. Reports of pandemic (H1N1) 2009 virus in skunks and anteaters also describe severe disease in those species (*2**,**3*).

In contrast, the infected black-footed ferret in our study had relatively mild clinical illness, consisting only of lethargy. This finding was surprising given recent experimental studies that reported the current pandemic (H1N1) 2009 virus was more pathogenic in domestic ferrets (*Mustela putorius furo*) than typical seasonal influenza viruses (*6*). However, several factors could have resulted in the low level of disease in this animal, such as prior immunity to influenza viruses or a low exposure dose. It is also possible black-footed ferrets are innately more resistant to influenza infection than domestic ferrets.

The origin of infection in these cases was not determined but was most likely an infected human. All animals had some level of contact with caretakers or veterinarians and were housed separately from other wildlife species. None of the potential human sources of virus had clinical signs before the animals became ill; however, influenza infections in humans can often be mild (*7*). Wild animals, such as opossums and skunks, that occasionally enter the zoological garden, represent another possible source. Good hygiene and husbandry practices used within the enclosures of the badger, binturong, and ferret failed to prevent infection, which suggests pandemic (H1N1) 2009 is efficiently transmitted to these species. Descriptions of infection in giant anteaters and cheetahs kept under similar conditions also support high transmissibility of influenza A viruses to animals, as do ongoing findings for swine (*3**,**4**,**8*).

Although ferrets are known to be susceptible to influenza A virus, to our knowledge, influenza in badgers and binturongs has not been reported. Badgers and binturong have been housed in zoological gardens for decades without incidence of influenza. Increased surveillance for influenza by the scientific community during the pandemic may have resulted in the novel recognition of infection in these species. Alternatively, the current pandemic (H1N1) 2009 virus may have a broader host range and stronger virulence than viruses in the past.

Pandemic (H1N1) 2009 was first detected in humans in March 2009 and reached pandemic levels by June of that year, rapidly establishing a rich pool for the development of genetic variants. Naturally acquired disease has now been described in 10 animal species, and experimental infection has been reported in an additional 2 animals (mice and cynomolgus macaques) (*9*). The ubiquity of pandemic (H1N1) 2009 and its ability to infect a diverse range of hosts is worrisome for the health of wildlife and for the possibility of creating additional reservoirs that could alter the evolution of subtype H1N1 viruses by applying varied selection pressures and establishing new ways of generating unique reassortant strains.
